# *Streptococcus suis* – The “Two Faces” of a Pathobiont in the Porcine Respiratory Tract

**DOI:** 10.3389/fmicb.2018.00480

**Published:** 2018-03-15

**Authors:** Désirée Vötsch, Maren Willenborg, Yenehiwot B. Weldearegay, Peter Valentin-Weigand

**Affiliations:** Institute for Microbiology, Center for Infection Medicine, University of Veterinary Medicine Hannover, Hannover, Germany

**Keywords:** *Streptococcus suis*, pathobiont, porcine respiratory tract, co-infections, respiratory infections

## Abstract

*Streptococcus (S.) suis* is a frequent early colonizer of the upper respiratory tract of pigs. In fact, it is difficult to find *S. suis*-free animals under natural conditions, showing the successful adaptation of this pathogen to its porcine reservoir host. On the other hand, *S. suis* can cause life-threatening diseases and represents the most important bacterial cause of meningitis in pigs worldwide. Notably, *S. suis* can also cause zoonotic infections, such as meningitis, septicemia, endocarditis, and other diseases in humans. In Asia, it is classified as an emerging zoonotic pathogen and currently considered as one of the most important causes of bacterial meningitis in adults. The “two faces” of *S. suis*, one of a colonizing microbe and the other of a highly invasive pathogen, have raised many questions concerning the interpretation of diagnostic detection and the definition of virulence. Thus, one major research challenge is the identification of virulence-markers which allow differentiation of commensal and virulent strains. This is complicated by the high phenotypic and genotypic diversity of *S. suis*, as reflected by the occurrence of (at least) 33 capsular serotypes. In this review, we present current knowledge in the context of *S. suis* as a highly diverse pathobiont in the porcine respiratory tract that can exploit disrupted host homeostasis to flourish and promote inflammatory processes and invasive diseases in pigs and humans.

## Introduction

*Streptococcus (S.) suis* is a commensal part of the respiratory microbiota of pigs, in particular of the tonsils and nasal cavities, but it can also cause highly invasive infections, such as meningitis, arthritis, endocarditis, bronchopneumonia, as well as septicemia and sudden death (Arends et al., 1984; Feng et al., 2014; Segura et al., 2016). Notably, though the colonization rate is up to 100%, clinical cases of *S. suis* infections, associated with meningitis, septicemia, or pneumonia, are by far less frequently reported (Goyette-Desjardins et al., 2014). *S. suis* is also considered an emerging zoonotic agent which can cause meningitis and sepsis in humans (Gottschalk et al., 2010). In contrast to swine, humans seem to be rarely colonized by *S. suis.* However, this remains to be studied in more detail and, therefore, human carrier rates (reported to be approximately 5% on average worldwide with respect to people in contact with pigs or pig products) may be underestimated (Strangmann et al., 2002; Goyette-Desjardins et al., 2014).

*Streptococcus suis* infections are known to be multi-factorial, unfavorable environmental conditions facilitate the development of disease. The nasopharynx is a reservoir niche for *S. suis* and various other (potentially) pathogenic microorganisms and commensals (Opriessnig et al., 2011). In this niche commensals can act as innocent bystander microbes, which inherently colonize the respiratory mucosa and can support other facultative pathogens to induce clinical disease. Those facultative pathogenic organisms are known as pathobionts. If pathogens play a dominant role in population changes of the microbiota and additionally manipulate the host response they are so-called keystone pathogens which can enhance the virulence of pathobionts leading to dysbiosis and inflammatory disease (Hajishengallis and Lamont, 2016). For *S. suis*, synergistic activities with other bacterial agents, such as *Pasteurella multocida* or *Mycoplasma hyopneumoniae*, as well as respiratory viruses like *porcine reproductive and respiratory syndrome virus* (PRRSV), *porcine circovirus type 2*, and *swine influenza virus* (SIV) (Fablet et al., 2011, 2012) may increase the risk of invasive infections (Meng et al., 2015). SIV and PRRVS are well-known keystone pathogens, since they pave the way for *S. suis* infections leading to severe respiratory symptoms and serious pneumonia (Thanawongnuwech et al., 2000; Lin et al., 2015; Meng et al., 2015).

**FIGURE 1:**
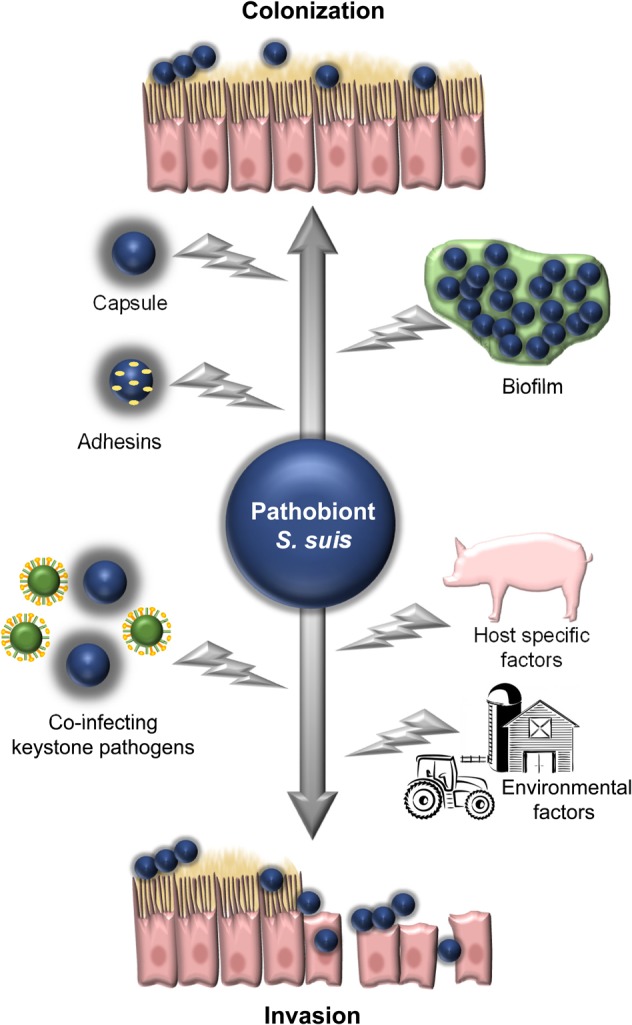
Illustration of the two phases (“faces”) of *S. suis* as a pathobiont in the respiratory tract. These are, firstly, as a colonization commensal bacterium and, secondly, as an invasive pathogen breaching different defense barriers. Some bacterial, host, and environmental factors, which contribute to the switch between both phases are depicted.

Nevertheless, interactions of *S. suis* with the mucosal immune system and evasion of innate immune defense mechanisms are crucial for induction of disease. *S. suis* has several immune evasion strategies, for example, expression of polysaccharide capsule (CPS) to prevent phagocytosis-dependent killing mechanisms (Segura et al., 2004; Chabot-Roy et al., 2006), or biofilm formation which may protect *S. suis* from antimicrobials (Grenier et al., 2009; Bojarska et al., 2016). Such features seem to play a role in virulence but may also be important for survival as a pathobiont. In this review, we focus on the role of *S. suis* as a typical respiratory pathobiont in swine, which possesses a highly invasive potential and causes severe infectious diseases in pigs and humans. In particular, we address the epidemiology of *S. suis* in pigs and humans, its diversity, and its two “faces” as a commensal and invasive pathogen (illustrated in **Figure 1**). Finally, we also include possible models to study host–pathobiont interactions in the respiratory tract. Since a number of excellent reviews on virulence mechanisms and virulence-associated factors have been published in recent years, we will include such mechanisms and factors only with respect to their potential role for the lifestyle of *S. suis* as a pathobiont.

## Epidemiology of *S. suis* in Pigs and Humans

*Streptococcus suis* is a widely distributed pathobiont and an emerging zoonotic pathogen. Its natural reservoir hosts are pigs (Lowe et al., 2011) and wild boars (Baums et al., 2007; Sanchez del Rey et al., 2014). Subclinical infected pigs play an important role in the epidemiology of *S. suis* as the main source of infection for other pigs and humans (Clifton-Hadley and Alexander, 1980). Susceptible pigs (especially weaning piglets) can suffer from meningitis, septicemia, pneumonia, endocarditis, or polyarthritis (Sanford and Tilker, 1982). Horizontal transmission via the respiratory tract due to nose-to-nose contact is the predominant route (Dekker et al., 2013), but vertical transmission from an infected sow to piglets via the genital tract during farrowing can also occur (Amass et al., 1997).

Humans can get infected with *S. suis* by eating raw or undercooked pork products (Fongcom et al., 2001; Gottschalk et al., 2010; Huong et al., 2014) or via cutaneous lesions when they get in contact with infected pigs or contaminated pork products (Wertheim et al., 2009). People in Asia are particularly affected because high-risk dishes, e.g., raw blood pudding, “*tiet canh*” (Huong et al., 2014), are common and the pig industry is more and more increasing while people are not aware of the risks of infection (Fulde and Valentin-Weigand, 2013). In Western countries only sporadic cases of human infections occur due to contact to infected pigs or raw pork meat (Goyette-Desjardins et al., 2014). In humans, *S. suis* causes mainly meningitis (Arends and Zanen, 1988) and septicemia [including streptococcal toxic shock-like syndrome/STSLS (Tang et al., 2006)], but cases of pneumonia, endocarditis, or peritonitis have also been reported (Huang et al., 2005). Some studies suggest that humans can also be healthy carriers of *S. suis* (Robertson and Blackmore, 1989; Elbers et al., 1999; Smith et al., 2008). Until end of December 2013 a total of 1642 human cases have been reported worldwide (Goyette-Desjardins et al., 2014), but this number has increased since then due to a high number of recent case reports and the high likelihood of misdiagnosis. Most of the human cases occurred in Asia (>90%), especially in Vietnam, Thailand, and China (Goyette-Desjardins et al., 2014). During the two outbreaks in China in 1998 and 2005, a total of 240 human cases were described (Tang et al., 2006; Yu et al., 2006).

Furthermore, *S. suis* can be found in the environment of slaughterhouses and wet markets (Ip et al., 2007; Ma et al., 2008; Nghia et al., 2011), particularly in Southeast Asia, which constitutes another source of infection for humans. In addition, *S. suis* was isolated from other animal species, such as rabbits, lambs, and dogs (Muckle et al., 2010; Sanchez del Rey et al., 2013; Muckle et al., 2014), though some of those had no close contact to pigs. These sources of infection should also be considered as a potential risk, especially since a few cases of human infections have been reported without any prior contact to pigs or pork products (Kerdsin et al., 2016).

## Diversity of *S. *suis**

*Streptococcus suis* is, genetically and phenotypically, a heterogeneous bacterial species. Strains belonging to different capsular serotypes or even to the same serotype differ from each other genetically (Blume et al., 2009; Gottschalk et al., 2013). For a comprehensive recent overview on the distribution of *S. suis* serotypes and genotypes, the reader is referred to a recent review by Goyette-Desjardins et al. (2014).

Multilocus sequence typing (MLST) is a method for genetic characterization, which allows to evaluate the epidemiology, the relation between different strains and virulent properties in more detail (Urwin and Maiden, 2003). King et al. (2002) have established a model of MLST for *S. suis* using seven different house-keeping genes (*cpn60, dpr, recA, aroA, thrA, gki*, and *mutS*). The nucleotide sequences of several alleles can be associated with each gene and the combination of those alleles of each isolate defines the sequence type (ST). Isolates with the same ST belong to the same clone. Occasionally, the same ST can comprise isolates with different serotypes and isolates with the same serotype could belong to different ST (King et al., 2002). In general, ST1 is mostly associated with clinical cases in both, pigs and humans, in Europe (Schultsz et al., 2012), Asia (King et al., 2002; Mai et al., 2008; Takamatsu et al., 2009), and Argentina (Callejo et al., 2016). ST7, which is responsible for the two large outbreaks in China in 1998 and 2005, seems to be endemic in China and Hong Kong (Ye et al., 2006; Li et al., 2010; Zhu et al., 2013). In North America, most of the isolated and analyzed serotype 2 strains from pigs and humans belong to ST25 and ST28 (Fittipaldi et al., 2011), the latter one has also been reported in Japan (Chang et al., 2006; Onishi et al., 2012) and other countries (Segura et al., 2017). ST101 to 104 is endemic in Thailand (Takamatsu et al., 2008, 2009; Kerdsin et al., 2011a), whereas ST20 was found only in humans in Europe [The Netherlands and France (Schultsz et al., 2012)].

So far, 35 serotypes based on the antigenicity of the capsular polysaccharides are known, but some of them have been suggested to belong to different bacterial species (Okura et al., 2016). Okura et al. (2013) developed a PCR which is able to detect all 35 serotypes but cannot distinguish serotypes 2 and 14 from serotypes 1/2 and 1, respectively. Especially these serotypes are commonly isolated from pigs (Wertheim et al., 2009). Additionally, there are many nontypable isolates, which do not agglutinate with any of the typing antisera (Messier et al., 2008) and which either belong to unknown encapsulated serotypes or non-encapsulated strains (Goyette-Desjardins et al., 2014). As reviewed recently by Goyette-Desjardins et al. (2014) the worldwide predominant serotype from diseased pigs is serotype 2 (27.9%), followed by serotypes 9 (19.4%) and 3 (15.9%). A total of 15.5% of the isolates were nontypable by serotyping (Goyette-Desjardins et al., 2014). The serotype distribution is different in healthy pigs, from which serotype 2 is less frequently isolated (Wisselink et al., 2000; Marois et al., 2007; Luque et al., 2010; Wang et al., 2013a). Unfortunately, there exist only few epidemiological data on *S. suis* in diseased pigs from those countries where most human cases are reported (Goyette-Desjardins et al., 2014). In some European countries, serotype 9 was more frequently found in diseased pigs and wild boars than serotype 2 (Vela et al., 2003; Tarradas et al., 2004; Luque et al., 2010; Schultsz et al., 2012; Sanchez del Rey et al., 2014), but until now no human serotype 9 cases have been reported. A recent study from Thailand revealed serotype 23 being the most prevalent in healthy pigs in Phayao Province, followed by serotypes 9, 7, and 2. Thirty-seven percent of the isolates was nontypable by multiplex PCR (Thongkamkoon et al., 2017). Another study from Northern Thailand (Chiang Mai Province) found mostly serotype 3 isolates in submaxillary glands of pig carcasses sold in wet markets (Wongsawan et al., 2015). A further study investigated samples from asymptomatic pigs from central Thailand and found mainly serotype 16 strains (Meekhanon et al., 2017). Taken together, *S. suis* serotype distribution differs worldwide, within a country, and even within the same region. Moreover, it was shown that pigs can be colonized by different serotypes at the same time (Flores et al., 1993). This raises the question whether the disease of a given animal is caused by a strain of a certain serotype or by interactions of several strains of different serotypes. Furthermore, it is unclear why some pigs in a herd get infected by a certain serotype while others do not (Higgins et al., 1990).

Worldwide, human cases are reported to be mainly due to serotype 2 (74.7%) and 14 (2.0%), both serotypes are equally involved in cases of meningitis (50–70%) and septicemia (20–25%) (Goyette-Desjardins et al., 2014). Only occasional cases were reported to be caused by serotypes 4, 5, 16, 21, 24, and 31 (Arends and Zanen, 1988; Nghia et al., 2008; Kerdsin et al., 2011b, 2016; Callejo et al., 2014; Gustavsson and Rasmussen, 2014; Hatrongjit et al., 2015; Taniyama et al., 2016). Most of the persons, who were infected by serotypes other than serotype 2, were suffering from a pre-existing liver cirrhosis (Kerdsin et al., 2011b; Taniyama et al., 2016) or other immunocompromising illnesses (Callejo et al., 2014). This suggests that those serotypes may be less virulent than serotype 2 strains. Callejo et al. (2016) reported that *S. suis* isolated from human cases in Argentina between 1995 and 2016 from cases of meningitis were caused by serotype 2 strains, except for one case caused by a serotype 5 isolate (Callejo et al., 2016). This is interesting because only few human cases have been reported from South America so far. One case of meningitis due to *S. suis* serotype 2 from Togo was reported in 2016 (Prince-David et al., 2016), which illustrates the emergence of this pathogen in a country, where it was previously unrecognized.

Although human cases occur only sporadically in Europe, the reported cases count for 8.5% of all human cases worldwide (Goyette-Desjardins et al., 2014). This may be explained by the fact that most of the European countries have a highly developed pig industry and the virulent serotype 2 can be found frequently in diseased pigs (Wisselink et al., 2000). In North America, only a few cases of human infections have been reported, although this country has the highest number of reports from diseased pigs (Goyette-Desjardins et al., 2014). One possible explanation may be that serotype 2 strains from North America are less virulent than Eurasian strains (Lachance et al., 2013).

## The Commensal “Face” of *S. suis*

Although the survival mechanism of *S. suis* as a pathobiont remains to be elucidated, it seems clear that the *S. suis* genome of approximately 2 Mbp encodes for a variety of enzymes, putative adhesins, and other factors, which enable it to colonize the host with other commensals (and pathogens). Here, we focus on bacterial factors and mechanisms, which most likely are important for the commensal life of *S. suis* in the respiratory tract, though these factors may also contribute to virulence (**Figure 1**, upper part).

The innate and adaptive immune mechanisms in the respiratory tract play a major role in pathogen recognition, processing, and elimination thereby maintaining tissue homeostasis (Whitsett and Alenghat, 2015). Mucociliary activity of ciliated epithelial cells is a major defense barrier encountered by microbes entering the host via the respiratory tract. However, some pathogens, including *S. suis*, have adapted to colonize the respiratory cilia. Thus, the initial step in colonization, bacterial adherence, is crucial for development of a carrier state (Brassard et al., 2004). First studies on adhesins of *S. suis* were published in the early 1990s (Haataja et al., 1993, 1994, 1996; Tikkanen et al., 1995, 1996). In recent years, we learnt much more about mechanisms of adherence and tissue tropisms of *S. suis*, though we still do not know precisely what adhesins are essential for infection.

Salivary glycoproteins in humans have terminal sialic acids, which are reported to serve as glycan receptor motifs. These motifs are commonly recognized by commensal streptococcal bacteria such as, e.g., *Streptococcus gordonii* (Takahashi et al., 1997; Takahashi et al., 2002; Loimaranta et al., 2005; Deng et al., 2014). A recent study by Chuzeville et al. (2017) revealed that *S. suis* serotype 2 and 9 strains express genes coding for multimodal adhesion proteins known as antigen I/II (AgI/II). In the presence of salivary glycoproteins, AgI/II leads to the aggregation of *S. suis*, adherence, and colonization of the upper respiratory tract of pigs. Especially in serotype 9, the AgI/II is reported to facilitate aggregation and biofilm formation, and these aggregated bacteria could be swallowed, but are protected from the low pH in the stomach, which may enhance colonization of the intestine (Chuzeville et al., 2017). *S*. *suis* also has an adhesin known as factor H-binding protein, Fhb (Pian et al., 2012; Roy et al., 2016; Zhang et al., 2016). Factor H is an abundant host protein in the plasma, which is responsible in protecting the host from excessive complement effects and maintains complement homeostasis [reviewed in de Cordoba and de Jorge (2008)]. Binding of *S. suis* to factor H by Fhb results in enhanced adherence of the bacteria to epithelial and endothelial cells. Fhb also protects *S. suis* from phagocytosis and complement mediated killing (Pian et al., 2012; Roy et al., 2016). Zhang et al. (2016) reported the structural domains involved in binding of Fhb to the host cell receptor glycolipid GbO3, which is abundantly expressed on endothelial cells and certain epithelial cells. For further details on *S. suis* adhesins involved in adhesion to epithelial cells, the reader is referred to a recent comprehensive review on initial steps of *S. suis* pathogenesis (Segura et al., 2016).

The most prominent structure of *S. suis* is the polysaccharide capsule, of which several different antigen types exist, as described above. Most likely, the capsule covers adherence-mediating surface components, but it does not completely inhibit adherence to host cells. Accordingly, some studies showed that the absence (or downregulation) of the capsule increases the exposure of adhesins and subsequent bacterial adherence (Salasia et al., 1995; Lalonde et al., 2000; Benga et al., 2004; Esgleas et al., 2005). The thickness of the capsule depends on the bacterial environment in its host niche. It has been reported that the capsule is thinner during colonization and invasion of the respiratory epithelium, possibly to expose adhesins for better attachment to the epithelial cells (Gottschalk and Segura, 2000). Tanabe et al. (2010) also reported that the capsule hinders adhesins and hydrophobic components of *S. suis*, which are responsible for biofilm formation. However, in the bloodstream, the thickness of the capsule is higher and this enables *S. suis* to escape phagocytosis (Smith et al., 1999a,b; Gottschalk and Segura, 2000; Segura et al., 2004; Roy et al., 2016). This underlines that the expression of the capsule needs to be controlled during colonization (and subsequent infection).

The expression of genes responsible for *S. suis* capsule synthesis is regulated by transcriptional regulators such as catabolite control protein A (CcpA) (Willenborg et al., 2011) and small RNAs (sRNAs) like sRNA rss04. According to Willenborg et al. (2011) depletion of *ccpA* gene in *S. suis* resulted in a phenotype similar to a non-encapsulated strain, whereas the *ccpA* mutant showed reduced capsule thickness and higher susceptibility to phagocytosis compared to the wild-type (WT) parental strain (Willenborg et al., 2011). In contrast, the sRNA rss04 has an opposite effect. Transmission electron microscopic analysis revealed that *S. suis* Δ*rss04* had a thicker capsule compared to the WT and complemented strains and, therefore, its presence appears to repress CPS production by downregulating the expression of *ccpA* (Xiao et al., 2017). Thus, most likely capsule synthesis and its coordinated regulation are very important for the colonization and survival of *S. suis* as a pathobiont.

Some microorganisms escape hostile environments by aggregation in the form of biofilms that enable them to persist and colonize tissues, resist clearance from host defense mechanisms and antimicrobials, and facilitate exchange of genetic information (Donlan and Costerton, 2002). *S. suis* is able to form biofilms which is controlled by quorum sensing. This is a signaling network regulated by *luxS* gene (coding for the enzyme *S*-ribosylhomocysteinase, LuxS), which has been found in virulent *S. suis* serotype 2 strains. It has been reported that LuxS plays an important role by its ability to enhance the biosynthesis of auto-inducer 2 (AI-2), adherence, biofilm formation, cell metabolism, and resistance to host immune responses and antimicrobial therapy (Zhu et al., 2002; Vendeville et al., 2005; Han and Lu, 2009; Wang et al., 2011b, 2013c, 2015). Biofilm production by *S. suis* is induced via the activity of fibrinogen-mediated cross bridging of *S. suis*. The presence of fibrinogen could stimulate the expression of adhesins thereby facilitating adherence of the bacteria to each other (Bonifait et al., 2008). Moreover, mucin, produced by goblet cells, may enhance biofilm formation and promote survival in nutrient-limited condition as reported for *Streptococcus mutans* (Mothey et al., 2014). Bacteria forming biofilms resist antimicrobials better than planktonic cells (Olson et al., 2002; Grenier et al., 2009). It has been reported that virulent strains of *S. suis* have a higher ability to produce biofilms than avirulent strains (Wang et al., 2011a). The same authors reported that the adherence of *S. suis* forming a biofilm to human pharyngeal epithelial (HEp-2) cells was lower than that of planktonic cells suggesting a reduced virulence of *S. suis* in the former stage. On the one hand, in biofilms bacterial metabolism and expression of virulence-associated genes is reduced; on the other hand, secreted toxins may be trapped in the polysaccharide matrix resulting in less tissue damage to the host (Wang et al., 2011a). This may explain why virulent strains of *S. suis* can also be harmless components of the respiratory microbiome.

## The Pathogenic “Face” of *S. suis*

As a facultative pathogenic bacterium, *S. suis* causes infectious diseases that are considered to be multifactorial, i.e., whether an initial infection remains subclinical or leads to clinical infection depends on several factors. It is long known that unfavorable environmental conditions such as overcrowding, poor ventilation and climatic conditions, poor hygiene status, high air pollution load, and other stressors correlate with an increasing clinical disease rate in pigs (Power, 1978; Sanford and Tilker, 1982; Chanter et al., 1993; Staats et al., 1997). Furthermore, host-specific factors, such as age, genetic background, and immunosuppression, influence disease development. Weaning piglets are most susceptible since protective maternal antibodies decline (Cloutier et al., 2003). Besides, pigs suffering from other bacterial and/or viral infections of the upper respiratory tract are more susceptible. In humans, especially advanced age and presence of pre-existing medical conditions that suppress the immune system are common predisposing factors for clinical *S. suis* infections (Arends and Zanen, 1988; Ma et al., 2008).

When (colonizing) *S. suis* encounters conditions that favor its replication, invasion, and evasion of immune control mechanisms, the opportunistic pathogen becomes pathogenic. This transition seems to depend on the individual strain and its equipment with virulence-(associated) factors, since only certain strains, geno-, and serotypes are isolated from diseased animals. In addition to the presence of virulence-related genes, their coordinated expression during infection is crucial for pathogenicity. Thus, host-, environment-, and pathogen-dependent factors are drivers of pathogenicity (**Figure 1**).

The respiratory tract can easily be colonized by environmental microorganisms which get access via direct contact or by aerosols. Thus, the upper airway tract harbors a complex and dynamic population of bacterial species including, e.g., *Haemophilus parasuis, M. hyopneumoniae, Actinobacillus pleuropneumoniae, Actinobacillus suis, P. multocida, Bordetella bronchiseptica*, and *S. suis*, as well as viruses like PRRSV, porcine circovirus type 2, and SIV (MacInnes et al., 2008; Opriessnig et al., 2011). Accordingly, porcine respiratory disease is often referred to as porcine respiratory disease complex due to its polymicrobial nature (Opriessnig et al., 2011).

The members of the respiratory microbiota differ in their intrinsic pathogenic potential and their role in shaping the population structure thereby building a mixture of non-pathogenic (accessory) commensal bacteria, which act as innocent bystander microbes or support pathogenic bacteria, and facultative pathogens known as pathobionts, such as *S. suis*. Moreover, keystone pathogens, sometimes also named master manipulators, play a dominant role in population changes, which may lead to subversion of the host immune system. This can affect the composition of the microbiota resulting in dysbiosis and increase of virulence of pathobionts, which then exploit the disrupted homeostasis for their invasion into deeper tissues (Hajishengallis and Lamont, 2016). For *S. suis*, PRRSV is considered to act as a keystone pathogen since PRRSV and *S. suis* coinfections in pig herds are frequently found (Schmitt et al., 2001) and co-infection of *S. suis* with PRRSV have been reported to enhance morbidity of *S. suis* infections (Thanawongnuwech et al., 2000; Feng et al., 2001; Auray et al., 2016). Although the precise mechanism by which PRRSV predisposes pigs to *S. suis* infection is unknown, recent studies showed that an altered innate immune system and exacerbating inflammatory responses are responsible for increasing the risk of *S. suis* infection in PRRSV-co-infected pigs. *In vitro* studies support the assumption that a decreased phagocytic activity by PRRSV-infected dendritic cells and porcine pulmonary macrophages may lead to a higher susceptibility to a subsequent *S. suis* infection (Auray et al., 2016). Furthermore, an epidemiological association between PRRSV in pigs and *S. suis* infections in pigs and humans was described (Hoa et al., 2013; Huong et al., 2016). To the best of our knowledge, there are no reports on associations of human viral respiratory infections with human *S. suis* infections.

Likewise, *S. suis* seems to be a pathobiont for infection by SIV. Experimental co-infections of pigs with SIV-*S. suis* revealed more severe clinical symptoms as well as more serious pathological changes and apoptosis of lungs compared to pigs mono-infected with either *S. suis* or SIV (Lin et al., 2015). Although very little is known about interactions between viral and bacterial pathogens and their role in the co-pathogenesis of respective diseases, in general virus-induced damage of the mucociliary barrier and a decreased immune response are considered to predispose pigs to secondary infections and pneumonia by opportunistic bacterial pathobionts (Fablet et al., 2011). Meng et al. (2015) found that SIV-facilitated adherence, colonization, and invasion of *S. suis* in a porcine precision-cut lung slices (PCLS) co-infection model was mediated by virus-induced impairment of the ciliary activity (Meng et al., 2015). Similarly, enhanced adherence of *S. suis* and direct binding in a capsule-dependent manner of *S. suis* to SIV- or SIV-infected cells were also found in co-infected newborn pig tracheal cells (Wang et al., 2013b; Wu et al., 2015). Thus, binding of *S. suis* to SIV-pre-infected cells appears to enable the bacterium to switch to an invasive pathogen. A further feature of SIV-*S. suis* co-infections seems to be an increased inflammatory response due to upregulation of inflammatory mediators like chemokines, interleukins, cell adhesion molecules, and eicosanoids, as it has been found in *in vitro* and *in vivo* experiments (Wang et al., 2013b; Dang et al., 2014; Lin et al., 2015). On the other hand, *S. suis* can also affect SIV infection. Lin et al. (2015) found increased viral loads in nasal swabs and lungs in co-infected pigs (Lin et al., 2015). Likewise, infection ability of SIV was enhanced after treatment with *S. suis* culture supernatants *in vitro*, most likely due to the proteolytic activity of a *S. suis* protease (Wang and Lu, 2008). In contrast, Wu et al. (2015) observed a negative effect on the growth capacity of SIV in *S. suis* co-infected cells (Wu et al., 2015).

Nevertheless, interactions of different microorganisms in the respiratory tract and their contribution to co-pathogenesis remain unclear. As suggested by Siqueira et al. (2017) the modes of actions of pathogens could be either additive or synergistic. Metatranscriptomic and metabolomic studies and appropriate infection models will surely help for better understanding of bacterial interactions and their roles in causing diseases or carrier states (Siqueira et al., 2017).

## *In Vitro* Models to Study Host–Pathobiont Interactions in the Respiratory Tract

As outlined above, we are just beginning to understand the interplay of commensals, pathobionts, and keystone pathogens in the respiratory tract. Studies to dissect these complex processes should be carried out in respective animal models, e.g., in pigs, or under conditions which most closely mimic *in vivo* conditions. Since animal experiments have limitations for several reasons, *ex vivo/in vitro* tissue cell culture models receive more and more attention. Two of such models based on primary porcine respiratory epithelial cells are air–liquid-interface (ALI) cultures and PCLS. Both have been shown to be suitable to study host–pathobiont interactions of *S. suis* in the porcine respiratory tract (Meng et al., 2015, 2016).

**FIGURE 2:**
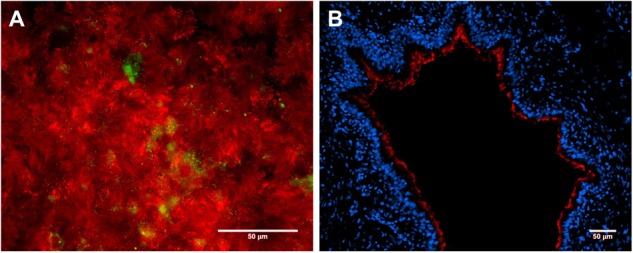
Primary porcine respiratory epithelial cell models to study host–pathobiont interactions in the respiratory tract. Immunofluorescence microscopy analysis of **(A)** primary porcine bronchial epithelial cells under air–liquid-interface (ALI) conditions after 3 weeks of differentiation and **(B)** a precision-cut lung slice (PCLS). Ciliated cells were stained by β-tubulin antibody (shown in red, **A + B**), mucin-producing cells were visualized by mucin 5-AC antibody (shown in green, **A**), and nuclei were stained by DAPI (shown in blue, **B**). Bars represent 50 μm.

For ALI cultures, primary porcine tracheal and bronchial epithelial cells (PTEC and PBEC) are isolated from swine lungs and cultured in a transwell system at ALI conditions for 4–5 weeks until the cells are well differentiated. Those well-differentiated respiratory epithelial cells build a pseudostratified epithelium, containing ciliated and mucin-producing cells (**Figure 2A**), and, therefore, represent the *in vivo* situation in the porcine respiratory tract. The expression of tight junction proteins and the development of a high *trans*-epithelial resistance indicate an epithelial barrier function (Prytherch et al., 2011). Human ALI culture systems have been proved suitable for modeling the respiratory tract by transcriptome analyses (Dvorak et al., 2011) and showing physiological responses to different pathogens (Krunkosky et al., 2007; Palermo et al., 2009). This cell culture model is particularly suitable to study the adherence and invasion of bacteria such as *S. suis* to well-differentiated respiratory epithelial cells and its effect on the epithelial barrier *in vitro* (Meng et al., 2016).

The advantage of the *ex vivo* PCLS model is that it preserves the structural and functional integrity of the lung, including the ciliary activity at the bronchiolar surface (**Figure 2B**), since those slices are pieces of lung tissue which can be kept in cell culture medium for several days. PCLS have been proved to be convincing alternatives to *in vivo* experiments for physiological, pharmacological, and toxicological investigations (Morin et al., 2013). This method allows to investigate the adherence, colonization, and invasion of bacteria like *S. suis* and to study microbial effects on bronchial epithelial cells, e.g., the ciliary motility and bronchus-constriction, by light microscopy. A limitation of PCLS is the restricted time of viability of the cells, making it less suitable for studying long-term effects.

Air–liquid-interface cultures and PCLS as well as further models, such as organoids from the respiratory tract, will have to be further improved, e.g., by including immune cells. Such models and respective imaging techniques will enable researchers in the future to dissect the complex interactions of microbes on mucosal surfaces with each other and the host, which will contribute to a better understanding of the role that pathobionts play in infection processes in the airway system.

## Author Contributions

PV-W developed the concept of the manuscript. DV, MW, YW, and PV-W wrote the manuscript.

## Conflict of Interest Statement

The authors declare that the research was conducted in the absence of any commercial or financial relationships that could be construed as a potential conflict of interest.
